# The 3p14.2 tumour suppressor ADAMTS9 is inactivated by promoter CpG methylation and inhibits tumour cell growth in breast cancer

**DOI:** 10.1111/jcmm.13404

**Published:** 2017-11-29

**Authors:** Bianfei Shao, Yixiao Feng, Hongbin Zhang, Fang Yu, Qianqian Li, Cui Tan, Hongying Xu, Jianming Ying, Lili Li, Dejuan Yang, Weiyan Peng, Jun Tang, Shuman Li, Guosheng Ren, Qian Tao, Tingxiu Xiang

**Affiliations:** ^1^ Chongqing Key Laboratory of Molecular Oncology and Epigenetics The First Affiliated Hospital of Chongqing Medical University Chongqing China; ^2^ The Second people's hospital of JingDe Zhen Jiangxi China; ^3^ The Sixth people's hospital of Chongqing Chongqing China; ^4^ Cancer Epigenetics Laboratory State Key Laboratory of Oncology in South China Department of Clinical Oncology Sir YK Pao Center for Cancer and Li Ka Shing Institute of Health Sciences The Chinese University of Hong Kong and CUHK Shenzhen Research Institute Hong Kong China; ^5^ Department of Pathology Cancer Hospital Peking Union Medical College & Chinese Academy of Medical Sciences Beijing China

**Keywords:** ADAMTS9, tumour suppressor, methylation, EGFR, TGFβ

## Abstract

Chromosome region 3p12‐14 is an important tumour suppressor gene (TSG) locus for multiple cancers. *ADAMTS9*, a member of the metalloprotease large family, has been identified as a candidate 3p14.2 TSG inactivated by aberrant promoter CpG methylation in several carcinomas, but little known about its expression and function in breast cancer. In this report, *ADAMTS9* expression and methylation was analysed in breast cancer cell lines and tissue samples. *ADAMTS9* RNA was significantly down‐regulated in breast cancer cell lines (6/8). After treating the cells with demethylation agent Aza and TSA,*ADAMTS9* expression was dramatically increased. Bisulphite genomic sequencing and methylation‐specific PCR detected promoter methylation, which was associated with decreased *ADAMTS9* expression. Hypermethylation was also detected in 130/219 (59.4%) of primary tumours but only in 4.5% (2/44) of paired surgical margin tissues. Ectopic expression of *ADAMTS9* in tumor cells induced significant growth suppression, cell cycle arrest at the G0/G1 phase, enhanced apoptosis and reduced cell migration and invasion. Conditioned culture medium from *ADAMTS9*‐transfected BT549 cells markedly disrupted tube formation ability of human umbilical vein endothelial cell (HUVEC) in Matrigel. Furthermore, *ADAMTS9* inhibited AKT signaling and its downstream targets (MDM2, p53, p21, p27, E‐cadherin, VIM, SNAIL, VEGFA, NFκB‐p65 and MMP2). In addition, we demonstrated, for the first time, that *ADAMTS9* inhibits AKT signaling, through suppressing its upstream activators EGFR and TGFβ1/TβR(I/II) in breast cancer cells. Our results suggest that *ADAMTS9* is a TSG epigenetically inactivated in breast cancer, which functions through blocking EGFR‐ and TGFβ1/TβR(I/II)‐activated AKT signaling.

## Introduction

Breast cancer (BC) is the leading cause of cancer‐related death in females between the ages of 20 and 59 in United States and China 1, 2. Each year, more than 1.15 million new cases are diagnosed worldwide 3. Similar to other tumour types, breast cancer is resulted from activation of oncogenes and inactivation of tumour suppressor genes (TSG). Methylation of the promoter plays a major role in gene regulation. Together with histone acetylation, promoter methylation presents a key mechanism in silencing TSG expression. During BC development and progression, a number of genes involved in DNA repair, cell cycle regulation, cell adhesion and signal transduction are silenced by aberrant methylation 4. Extensive studies over the past decade have established aberrant DNA methylation as one of the most common molecular abnormalities in BC 3.

A disintegrin‐like and metalloprotease with thrombospondin type 1 motif 9 (*ADAMTS9*) encodes a member of the large family of ADAMTS that are implicated in tissue morphogenesis, pathophysiological remodelling, inflammation and vascular biology 5. Distinct from the well‐characterized metalloprotease family, a disintegrin and metalloprotease (ADAM), ADAMTS proteins are characterized by having the thrombospondin type 1 repeats (TSR) domains, which may be important for binding the extracellular matrix and suppressing angiogenesis 6, 7. In recent decades, several studies have reported the direct involvement of ADAMTS family proteins in tumour progression and metastasis 8. ADAMTS1 was initially characterized as an anti‐angiogenic molecule that exerts inhibitory effects on tumours 9. Recent methylation studies led to the identification of another family member, ADAMTS18, that plays a crucial role in several human cancers including nasopharyngeal carcinoma (NPC) and esophageal squamous cell carcinoma (ESCC) 10.

*ADAMTS9* was identified as a critical candidate TSG in ESCC in 2007. The *ADAMTS9* gene was located to the 1.61 Mb tumour suppressive critical region in chromosome 3p14.2 and showed a significant expression down‐regulation that was associated with promoter hypermethylation in both ESCC cell lines and primary esophageal tumour tissue 11. Subsequently, ADAMTS9 was suggested as a novel tumour suppressor based on its remarkable activities in inducing apoptosis and inhibiting cell proliferation and angiogenesis in nasopharyngeal, gastric, colorectal, pancreatic and cervical cancers 12, 13, 14, 15, 16. Notably, expression of *ADAMTS9* was significantly down‐regulated or lost in all these cancer types by promoter hypermethylation 12, 13, 14, 16. In addition, the expression of ADAMTS9 antisense RNA 2 (ADAMTS9‐AS2) is negatively correlated with DNA methyltransferase‐1 (DNMT1) 17.

The expression and function of *ADAMTS9* in breast cancer was not well studied as there are few reports 18. The effect of *ADAMTS9* on breast carcinogenesis is yet to be established. We investigated the hypothesis that promoter methylation plays the vital role in *ADAMTS9 expression* regulation, which underlies a major mechanism for breast cancer development and progression.

## Materials and methods

### Cell culture and tumour samples

The panel of breast tumour cell lines used in this study includes BT549, MCF‐7, T47D, MDA‐MB‐231, MDA‐MB‐468, SK‐BR‐3, YCC‐B1 and YCC‐B3. YYC‐B1 and YCC‐B3 were provided by Dr Sun Young Rha (Yonsei Cancer Center, Korea). The human mammary epithelial cell line, HMEpC (Applied Biosystems, Foster City, CA, USA), was used as a control. Human umbilical vein endothelial cells (HUVECs) were purchased from American Type Culture Collection (ATCC). Cells were cultured as described previously 19. EGF treatment was carried out by treating cells with recombinant human EGF protein (50 ng/ml, Invitrogen Corporation, Carlsbad, CA, USA) for 40 min.; then, the cells were harvested. TGF‐β1 (recombinant Human TGF‐β1, 100‐21C, PeproTech, Rocky Hill, NJ, USA) was used at a final concentration of 1 ng/ml for treating cells for 24 hrs. Cells were treated with 5 μM of LY2109761 (selective TGF‐β receptor type I/II dual inhibitor, Selleck, Houston, USA) for 24 hrs.

Normal human adult breast tissue RNA samples were purchased from Stratagene (La Jolla, CA, USA) or Millipore Chemicon (Billerica, MA, USA). Breast tumour and paired surgical margin tissues were obtained after surgical procedures from the First Affiliated Hospital of Chongqing Medical University. All samples were subjected to histologic diagnosis by pathologists. Clinical information including age, tumour grade, tumour size, ER status, PR status, HER2 status and p53 status was obtained for the majority of tumour cases. Tumour grading was achieved by staining with haematoxylin and eosin (H&E). Informed consent was obtained from patients for acquisition of tissue specimens. The Ethics Committee of the First Affiliated Hospital of Chongqing Medical University approved this study [Approval notice: 20120307].

### Treatment of cells with Aza and TSA

Cell lines were treated as described previously 19, 20. Briefly, Cell lines were treated with 10 mmol/l 5‐Aza‐dC (Sigma‐Aldrich, St Louis, MO, USA) for 3 days and further treated with 100 nmol/l trichostatin A (TSA, Cayman Chemical Co., Ann Arbor, MI) for an additional 24 hrs.

### Semi‐quantitative reverse transcription‐PCR

Total RNA was isolated using the TRIzol^®^ Reagent (Invitrogen Corporation). Reverse transcription polymerase chain reaction (RT‐PCR) was performed as described previously using Go‐Taq polymerase (Promega, Madison, WI, USA) and the GeneAmp RNA PCR system (Applied Biosystems), with glyceraldehyde 3‐phosphate dehydrogenase (*GAPDH*) as the control 21. The primer pairs used are listed in Table 1.

**Table 1:** List of primers used in this study

PCR	Primer	Sequence (5′‐3′)	Product size (bp)	PCR cycles	Annealing temperature (°)
RT‐PCR	ADAMTS9‐F	CATGCAGTTTGTATCCTG	218	32	55
	ADAMTS9‐R	GCGTTCTTTTGAAGTGGACG			
	GAPDH‐F	TCCTGTGGCATCCACGAAACT	315	23	55
	GAPDH‐R	GAAGCATTTGCGGTGGACGAT			
qRT‐PCR	p53‐F	TCAACAAGATGTTTTGCCAACTG	118	40	60
	p53‐R	ATGTGCTGTGACTGCTTGTAGATG			
	Ecad‐F	TGCCCAGAAAATGAAAAAGG	200	40	60
	Ecad‐R	GTGTATGTGGCAATGCGTTC			
	VIM‐F	GACCAGCTAACCAACGACAA	150	40	60
	VIM‐R	GTCAACATCCTGTCTGAAAGAT			
	SNAIL‐F	GAGGCGGTGGCAGACTAG	159	40	60
	SNAIL‐R	GACACATCGGTCAGACCAG			
	VEGFA‐F	CACACAGGATGGCTTGAAGA	136	40	60
	VEGFA‐R	AGGGCAGAATCATCACGAAG			
	TGFβ1‐F	AATTGAGGGCTTTCGCCTTAG	87	40	60
	TGFβ1‐R	CCGGTAGTGAACCCGTTGAT			
	TGFβR1‐F	ATGGGCTCTGCTTTGTCTCT	254	40	60
	TGFβR1‐R	AGCAATGACAGCTGCCAGTT			
	EGF‐F	GCTGCTCACTCTTATCATTCTG	234	40	60
	EGF‐R	CATGATCACTGAGACACCAG			
	EGFR‐F	TTCCTATGCCTTAGCAGTCTTAT	142	40	60
	EGFR‐R	GATGCTCTCCACGTTGCACAG			
	β‐actin‐F	GTCTTCCCCTCCATCGTG	113	40	60
	β‐actin‐R	AGGGTGAGGATGCCTCTCTT			
MSP	ADAMTS9‐M1	TTTTTCGTTTTTTTTTGTTCGTTC	114	40	60
	ADAMTS9‐M2	AAACTAAACCGCTCGAACCG			
	ADAMTS9‐U1	GTTTTTTGTTTTTTTTTGTTTGTTT	117	40	58
	ADAMTS9‐U2	AAAAACTAAACCACTCAAACCA			
BGS	ADAMTS9‐BGS1	GTATTTGAGAGGTTGTGGATT	390	40	60
	ADAMTS9‐BGS2	CCTCCTACCCTCCTTAACTA			

### Methylation‐specific PCR and bisulphite genomic sequencing

Genomic DNA was extracted from cell pellets and tissues using the QIAamp DNA Mini Kit (QIAGEN, Hilden, Germany). DNA was chemically modified with 2.4 mol/l sodium metabisulphite for 4 hrs as described previously 21. At present, bisulphite sequencing (BS) has been known as the ‘gold standard’ for detecting DNA methylation 22, 23. Methylation‐specific PCR (MSP) and BS were carried out according to previous methods 24, 25. Bisulphite‐treated DNA was amplified with the *ADAMTS9* methylation‐specific primers and unmethylation‐specific primers (Table 1), respectively, using AmpliTaq‐Gold DNA Polymerase (Applied Biosystems). MSP primers were previously assessed to ensure specific amplification of bisulphite‐treated DNA. For BS analysis, bisulphite‐treated DNA was amplified with a pair of BS primer (Table 1) specific for CpG islands of the *ADAMTS9* promoter, which contains 45 CpG sites and spans the region of MSP analysis. Amplified products were cloned into the pCR4‐Topo vector (Invitrogen Corporation). Clones containing 8–10 colonies were randomly selected for sequencing (Beijing Genomics Institute, Beijing, China).

### Quantitative reverse transcription polymerase chain reaction (qRT‐PCR) analysis

qRT‐PCR was carried out on the ABI 7500 Real‐Time PCR System (Applied Biosystems) using Maxima SYBR Green/ROX qPCR Master Mix (MBI Fermentas, St. Leon‐Rot, Germany). The primer pairs used are listed in Table 1. All primers were designed to span exon–exon junctions and allowed specific amplification at 60°C through melt curve analysis and 2% agarose gel electrophoresis (data not shown), according to the Minimum Information for Publication of Quantitative Real‐Time PCR Experiments (MIQE) guidelines 26. Thermal cycling conditions were 95°C for 30 sec., followed by 5 sec. at 95°C, and 1 min. at 60°C for 40 cycles. Gene expression was defined based on the threshold cycle (Ct), and the relative expression levels were calculated using the 2‐ΔΔCt method. Relative expression levels of *ADAMTS9* in tissues were standardized to *ACTB* levels.

### ADAMTS9 overexpression in BT549 and SK‐BR‐3 cells

The expression of ADAMTS9 is decreased or silenced in six breast cancer lines. We chose BT549 and SK‐BR‐3 for further study. For ectopic ADAMTS9 expression, pCEP4‐ADAMTS9 with full‐length wild‐type *ADAMTS9* cDNA was used (originally from Dr Suneel S Apte, Department of Biomedical Engineering, Lerner Research Institute, Cleveland, OH, USA) 27. pCEP4 plasmid was purchased from Invitrogen (Invitrogen; Thermo Fisher Scientific, Inc. USA). pCEP4‐ADAMTS9 or pCEP4 was transfected into 80% confluent BT549 and SK‐BR‐3 cells using Lipofectamine 2000 reagent (Invitrogen Corporation) according to the manufacturer's protocol. After transfection, the cells were grown in non‐selective growth medium for 48 hrs. The medium was replaced with selection medium containing Hygromycin B (Sigma‐Aldrich, St Louis, MO, USA), 100 μg/ml for BT549 and 25 μg/ml for SK‐BR‐3, for 14 days. Total RNA and protein were extracted from transfected cells and analysed *via* RT‐PCR and Western blot, respectively, to confirm stable overexpression of *ADAMTS9*.

### Colony formation assay

BT549 cells stably expressing *ADAMTS9* or vector were seeded in a six‐well plate at 500 cells per well and allowed to grow for 7 days. SK‐BR‐3 cells stably expressing *ADAMTS9* or vector were seeded in a six‐well plate at 3000 cells per well and allowed to grow for 14 days. Surviving colonies (>50 cells/colony) were fixed with 4% paraformaldehyde and counted after staining with Gentian Violet (ICM Pharma, Singapore). All experiments were performed in triplicate.

### Cell proliferation assay

Cell proliferation was assessed using the CellTiter 96^®^ Aqueous One Solution Reagent (Promega) according to the manufacturer's protocol. Stable *ADAMTS9* or control vector‐expressing cells (BT549 and SK‐BR‐3) were seeded in 96‐well plates (2000 cells per well) with 100 μl complete medium and cultured for 24, 48 or 72 hrs, followed by incubation with 100 μl/well medium containing 20 μl CellTiter 96^®^ Aqueous One Solution reagent at 37°C for 2 hrs. Absorbance was measured at 490 nm using a microplate reader (Multiskan MK3; Thermo Fisher Scientific, former Fermentas, Schwerte, Germany). Data were obtained from three independent cultures, and each experiment was repeated three times.

### Flow cytometric analysis

For cell cycle assay, BT549 and SK‐BR‐3 cells were cultured in six‐well plates and grown overnight. Cultures were then transfected with pCEP4‐ADAMTS9 or pCEP4 using Lipofectamine 2000 reagent. After 48 hrs, cells were collected, digested with trypsin and centrifuged at 800 r.p.m. for 6 min., washed twice with phosphate‐buffered saline (PBS) and fixed in ice‐cold 70% ethanol overnight at 4°C. Next, cells were stained with 50 μg/l propidium iodide (PI; BD Pharmingen, San Jose, CA, USA) for 30 min. at 4°C in the dark. Cell sorting was performed using a FACSCalibur machine (BD Pharmingen, Franklin Lakes, NJ, USA), and data were analysed with ModFit 3.0 software (Verity Software House, Topsham, ME, USA).

Apoptosis was assessed using Annexin V‐fluorescein isothiocyanate (FITC; BD Pharmingen) and PI staining, in keeping with the manufacturer's protocol. Data were analysed with CellQuest™ Pro (BD Biosciences).

### Wound healing assay

Cells were seeded into six‐well plates and allowed to grow until confluence. A linear scratch ‘wound’ was created onto the cell monolayer with a sterile 10 μl tip. Cells were washed with PBS and cultured in serum‐free medium. Microscopic images (100× magnification, Nikon, Japan) of cells were taken every 24 hrs and evaluated based on the zero line of the linear ‘wound’. The experiments were performed in triplicate.

### Transwell cell migration assay

Transwell chambers (8 μm pore size; Corning, Corning, NY, USA) were employed to assess cell migration ability as described previously 19, 28. Cells were collected, washed twice in serum‐free medium and added to the upper chamber (2 × 10^4^ BT549 cells and 6 × 10^4^ SK‐BR‐3 cells). The lower chamber contained 700 μl migration‐inducing medium with 10% foetal bovine serum (FBS). After incubation (BT549 for 24 hrs, SK‐BR‐3 for 48 hrs), cells were fixed with 4% paraformaldehyde for 30 min. and stained for 30 min. with crystal violet. Non‐migratory cells on the upper side of the filter were wiped off using Q‐tips. Migrated cells were counted at 100× magnification under a microscope.

### Transwell cell invasion assay

Transwell chambers (8 μm pore) coated with Matrigel (BD Biosciences Discovery Labware) were used for the invasion assay. Cells diluted with serum‐free medium (2 × 10^4^ BT549 cells and 6 × 10^4^ SK‐BR‐3 cells) were seeded into the upper wells of pre‐coated transwell chambers. Lower wells of the chambers were filled with 700 μl medium containing 10% FBS. After incubation (BT549 for 24 hrs, SK‐BR‐3 for 48 hrs), cells were fixed with 4% paraformaldehyde for 30 min., followed by staining for 30 min. with crystal violet. Q‐tips were used for wiping off cells on the upper side of the filter. The invaded cells were counted under a microscope at 100× magnification.

### Tube formation assay

Conditioned media were collected by incubating pCEP4‐ADAMTS9 and vector‐transfected BT549 cells without serum for 24 hrs. The 96‐well plate was coated with 50 μl Matrigel (Millipore) after thawing on ice. Each well was incubated at 37°C for 30 min. to allow Matrigel polymerization. A total of 10^4^ HUVEC cells were seeded into each well and incubated with 200 μl conditioned media from ADAMTS9 and vector‐alone transfectants plus 1% FBS. Cells were incubated for 8 hrs to allow the formation of tube‐like structures. Tube‐like structures were counted under a microscope at 200× magnification. Image analysis of tube length was carried out using ImageJ software (NIH, MD, USA).

### Western blot

Cells were lysed using protein extraction reagent (Thermo Scientific) containing the protease inhibitor phenyl methane sulfonyl fluoride (PMSF) and a phosphatase inhibitor cocktail (Sigma‐Aldrich). Total protein concentrations were measured using the BCA protein assay (Thermo Scientific). Lysates were separated *via* sodium dodecyl sulphate/polyacrylamide gel electrophoresis (SDS‐PAGE) and transferred onto polyvinylidene fluoride (PVDF) membranes for antibody incubation. Membranes were incubated with blocking buffer (PBS with 5% non‐fat milk and 0.1% Tween 20) followed by the appropriate primary and secondary antibodies and visualized using the enhanced chemiluminescence (ECL) detection kit (Thermo Scientific). The following primary antibodies were used: ADAMTS9 (1:1000, Santa Cruz Biotechnology, Dallas, Texas, USA, sc‐21500), MDM2 (1:1000, Santa Cruz Biotechnology, sc‐965),p53 (1:1000, Santa Cruz Biotechnology, sc‐126), p21 (1:1000, Cell Signaling Technology, Danvers, MA, USA #2947), p27 (1:1000, Cell Signaling Technology, #3686), cleaved caspase 9 (1:1000, Cell Signaling Technology, #9501), caspase 3 (1:1000, Cell Signaling Technology, #9662), cleaved caspase 3 (1:1000, Cell Signaling Technology, #9664), AKT (1:1000, Cell Signaling Technology, #4691), phospho‐AKT (Ser473) (1:1000, Cell Signaling Technology, #4060), SNAIL (1:1000, Cell Signaling Technology, #3879), E‐Cadherin (1:1000, Abcam, Cambridge, MA, USA ab40772), VIM (1:1000, Santa Cruz Biotechnology, sc‐965), MMP2 (1:1000, Abcam, ab86607), VEGFA (1:1000, Origene, TA500042), phospho‐VEGF Receptor 2 (Tyr1175) (1:1000, Cell Signaling Technology, #2478),EGFR (1:1000, Abcam, ab32077), phospho‐EGFR (Y1068) (1:1000, Abcam, ab32430), phospho‐NFκB p65 (Ser536) (1:1000, Cell Signaling Technology, #3033) and β‐actin (1:1000, Abcam, Ab8226). Anti‐rabbit IgG and antimouse IgG horseradish peroxidase conjugate secondary antibodies were from Cell Signaling Technology; anti‐goat IgG horseradish peroxidase conjugate secondary antibodies were from Liankebio (RAG007).

### Statistical analyses

Statistical analyses for clinicopathological features of *ADAMTS9* methylation in breast cancer were performed using the chi‐square test and Fisher's exact test to determine the *P* value. Statistical analyses for qRT‐PCR analysis, colony formation assay, cell proliferation assay, flow cytometric analysis, wound healing assay, transwell assay and tube formation assay were performed using Student's *t*‐test. Differences between two groups were scored for statistical significance (*P < 0.05, **P < 0.01, ***P < 0.001, mean ± S.D.)

## Results

### ADAMTS9 expression is down‐regulated in breast cancer; methylation of ADAMTS9 CGI contributes to its down‐regulation in BC cell lines

**Figure 1:**
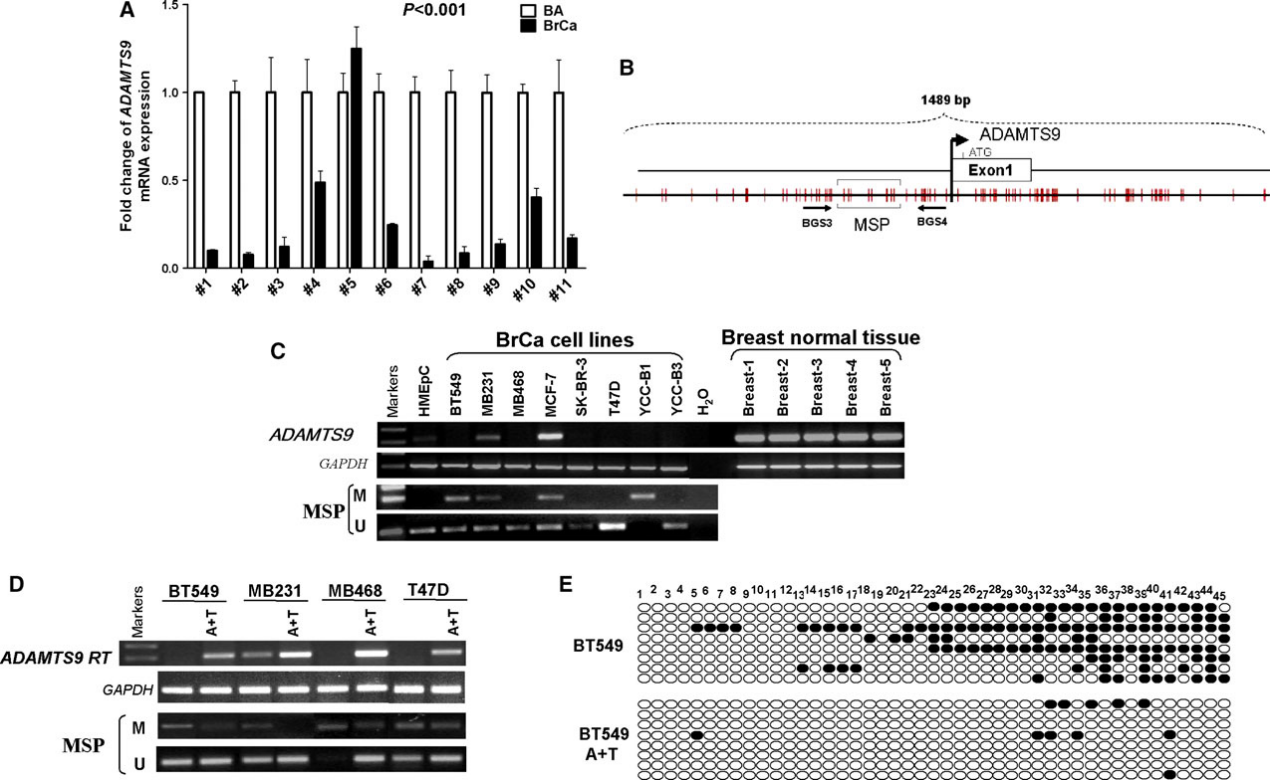
The expression and methylation status of ADAMTS9 in normal mammary tissues and breast cancer cell lines. (**A**) ADAMTS9 expression levels in paired breast tumour tissues and surgical margin tissues were detected by qPCR. BA: breast tumour adjacent tissues; BrCa: breast cancer tissues. (**B**) Schematic structure of the ADAMTS9 promoter CpG island (CGI). The white rectangle represents exon 1, and the CpG sites in the CGI are indicated with short black lines. (**C**) Expression of ADMTS9 in breast cancer cell lines and breast normal tissues, and the methylation status of ADAMTS9 in breast cancer cell lines and HMEpC cells. (**D**) Pharmacological demethylation of the ADAMTS9 CGI by Aza (A) with TSA (T) induced its expression. ADAMTS9 expression before and after drug treatment was determined by RT–PCR, and demethylation was confirmed by MSP. (**E**) Bisulphite genomic sequencing confirmed A +T treatment could inhibit methylation of the ADAMTS9 promoter.

We first examined the expression levels of *ADAMTS9* in 11 pairs of BC tissues and surgical margin tissues by qRT–PCR. The results showed that 10 of 11 (90.1%) tumours had lower *ADAMTS9* mRNA expression compared to their paired surgical margin tissues (Fig. 1A, *P* < 0.001). Consistently, *ADAMTS9* was highly expressed in normal human mammary epithelial cell lines (HMEpC), but greatly reduced or completely silenced in multiple breast carcinoma cell lines (6/8, 75%; Fig. 1C). A typical CGI CpG island (CGI) was identified in the *ADAMTS9* gene near exon 1 using the CGI Searcher (http://ccnt.hsc.usc.edu/cpgislands2) according to three parameters: GC content ≥ 55%, Obs CpG/Exp CpG ≥ 0.65 and length ≥ 500 bp (Fig. 1B), suggesting *ADAMTS9* is sensitive to methylation‐mediated silencing. To ascertain whether the down‐regulation of *ADAMTS9* is correlated with promoter hypermethylation, we analysed the methylation status of *ADAMTS9* CGI with MSP in eight breast carcinoma cell lines. As expected, *ADAMTS9* CGI was methylated in two of six cell lines with concomitant silencing or reduction of gene expression (Fig. 1C). In contrast, *ADAMTS9* CGI methylation was not detected in HMEpC (Fig. 1C), implying that this phenomenon occurs specifically in tumours.

To further illuminate if *ADAMTS9* silencing is directly regulated by CGI methylation, *ADAMTS9* mRNA levels in tumour cells were compared before and after treatment with 5‐aza‐dC (DNA methyltransferase inhibitor) and TSA (histone deacetylase inhibitor). Treatment with the drugs induced significant *ADAMTS9* expression (Fig. 1D), whereas the CGI was definitely demethylated in the presence of 5‐aza‐dC and TSA (Fig. 1D). The results collectively revealed a direct link between CpG methylation and *ADAMTS9* silencing and confirmed the pathway to CGI methylation‐mediated down‐regulation of *ADAMTS9*. We further examined the methylation status by BS analysis of 45 CpG sites including those examined using MSP within the *ADAMTS9* CGI. The results showed methylation of the majority of CpG sites in BT549 cells, which was removed after treating the cells with Aza and TSA (Fig. 1E), further confirming tumour‐specific methylation of the *ADAMTS9* CGI.

### Methylation of ADAMTS9 CGI in primary carcinomas

**Figure 2:**
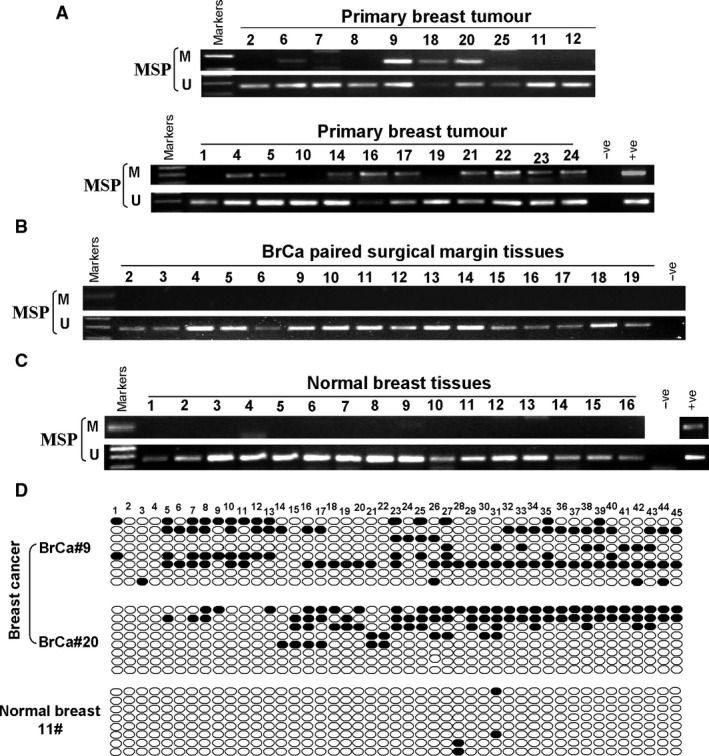
Promotor methylation status of ADAMTS9 in breast tumour tissues. (**A**) Representative images of methylation of the ADAMTS9 promoter in breast tumour tissues. (**B**) Methylation of ADAMTS9 in paired surgical margin tissues. (**C**) Methylation of ADAMTS9 in normal breast tissues. (**D**) Methylation status of the ADAMTS9 promoter in breast cancer tissues was significantly higher than that in normal breast tissues, confirmed by BGS. M, methylated; U, unmethylated.

We then investigated the status of *ADAMTS9* CGI methylation in BC and paired surgical margin tissues using normal tissue samples as controls. The results showed that the CGI methylation of *ADAMTS9* in breast cancer tissue was much higher than that in paired margin tissue and normal breast tissue (Fig. 2A–C). *ADAMTS9* methylation was detected in 130/219 (59.4%) of BC tissues, and 4.5% (2/44) of paired adjacent tissues, but not in normal epithelial tissues (Table 2), highlighting the relevance of tumour‐specific *ADAMTS9* methylation. Furthermore, we randomly selected one normal and two breast cancer tissue samples for BS (Fig. 2D). The results confirmed that hypermethylation of CGI is associated with low *ADAMTS9* expression in breast carcinoma.

**Table 2:** Methylation status of the *ADAMTS9* promoter in primary breast tumours

Samples	*ADAMTS9* promoter		Frequency of methylation
	Methylation	Unmethylation	
BrCa (*n* = 219)	130	89	59.4%
BA (*n* = 46)	2	44	4.5%
BN (*n* = 16)	0	16	0%

BrCa, breast cancer; BA, breast cancer adjacent (surgical margin tissues); BN, breast normal tissues.

Next, we analysed the relationship between *ADAMTS9* methylation and clinicopathological features of BC patients. The feature parameters include age, tumour size, tumour grade, lymph node metastasis, oestrogen receptor (ER), progesterone receptor (PR), HER2 status and p53 expression status. But, no visible correlation of *ADAMTS9* methylation with clinicopathological features was observed (Table 3).

**Table 3:** Clinicopathology features of *ADAMTS9* methylation in breast cancer

Clinicopathological features	Number (*n* = 219)	ADAMTS9 promoter methylation status		*P* value
		Methylated	Unmethylated	
Age				0.999
≤40	32	19 (59.4%)	13 (40.6%)	
>40	187	111 (59.4%)	76 (40.6%)	
Grade				0.312
1	13	8 (61.5%)	5 (38.5%)	
2	149	91 (61.1%)	58 (38.9%)	
3	24	10 (41.7%)	14 (58.3%)	
unknown	33	21 (63.6%)	12 (36.4%)	
Tumour size				0.079
≤2.0 cm	99	59 (59.6%)	40 (40.4%)	
>2.0 cm ≤ 5.0 cm	99	63 (63.6%)	36 (36.4%)	
>5.0 cm	18	8 (44.4)	10 (55.6%)	
unknown	3	0 (0.0%)	3 (100.0%)	
Lymph node metastasis				0.446
Positive	98	54 (55.1%)	44 (44.9%)	
Negative	117	74 (63.2%)	43 (36.8%)	
unknown	4	2 (50.0%)	2 (50.0%)	
ER status				0.943
Positive	122	73 (59.8%)	49 (40.2%)	
Negative	86	51 (59.3%)	35 (40.7%)	
unknown	11	6 (54.5%)	5 (45.5%)	
PR status				0.939
Positive	98	58 (59.2%)	40 (40.8%)	
Negative	110	66 (60.0%)	44 (40.0%)	
unknown	11	6 (54.5%)	5 (45.5%)	
HER2 status				0.030
Positive	13	7 (53.8%)	6 (46.2%)	
Negative	89	44 (49.4%)	45 (50.6%)	
unknown	117	79 (67.5%)	38 (32.5%)	
p53 expression				0.535
Positive	113	67 (59.3%)	46 (40.7%)	
Negative	83	47 (56.6%)	36 (43.4%)	
unknown	23	16 (69.6%)	7 (30.4%)	

### Ectopic ADAMTS9 expression inhibits proliferation and colony formation in breast carcinoma cells

**Figure 3:**
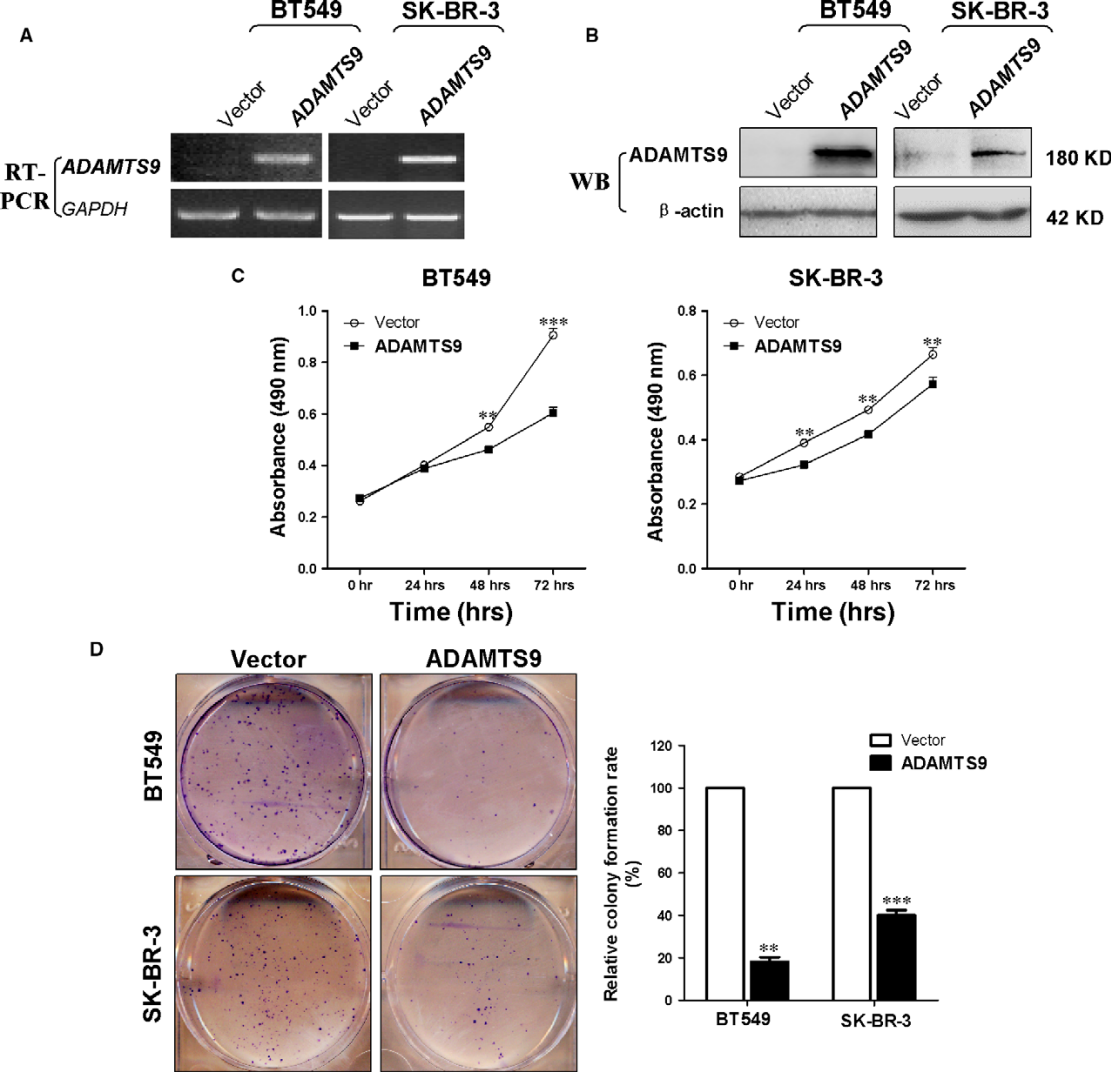
Ectopic ADAMTS9 inhibits cell proliferation and colony formation ability of BT549 and SK‐BR‐3 cells. (**A**) RT‑PCR confirmation of stable ectopic transcript expression of ADAMTS9 in transfected BT549 and SK‐BR‐3 cells. (**B**) Western blot confirmation of stable ectopic protein expression of ADAMTS9 in transfected BT549 and SK‐BR‐3 cells. (**C**) Cell proliferation curve was inhibited by ADAMTS9 in BT549 and SK‐BR‐3 cells. (**D**) Ectopic ADAMTS9 inhibited colony formation in BT549 and SK‐BR‐3 cells. Number of colonies in the vector‑transfected cell lines was set to 100%, values are presented as the mean ± S.D. **P < 0.01, ***P < 0.001.

To assess the effects of ectopic *ADAMTS9* expression on proliferation of breast carcinoma cells, the expression vector incorporating full‐length *ADAMTS9* was transfected into breast tumour cell lines, BT549 and SK‐BR‐3, and selected with Hygromycin B for 14 days to construct cell lines stably expressing *ADAMTS9*. Cells transfected with the empty vector pCEP4 were used as a control. Expression levels of transfected cells were determined *via* RT‐PCR and Western blot (Fig. 3A and B). The effect of *ADAMTS9* on cell proliferation was evaluated. Notably, ectopic expression of *ADAMTS9* in BT549 and SK‐BR‐3 cells significantly inhibited proliferation (*P* < 0.01, Fig. 3C). Meanwhile, colony formation of transfected cells was performed. In cells transfected with *ADAMTS9*, colony formation was significantly reduced, compared to control cells (reduced to 18% of control in BT549, *P* < 0.01, and 40% of control in SK‐BR‐3, *P* < 0.001, Fig. 3D), suggesting an inhibitory effect of *ADAMTS9* on proliferation in breast cancer cells.

### ADAMTS9 arrests the cell cycle at the G0/G1 phase and induces apoptosis in breast cancer cells

**Figure 4:**
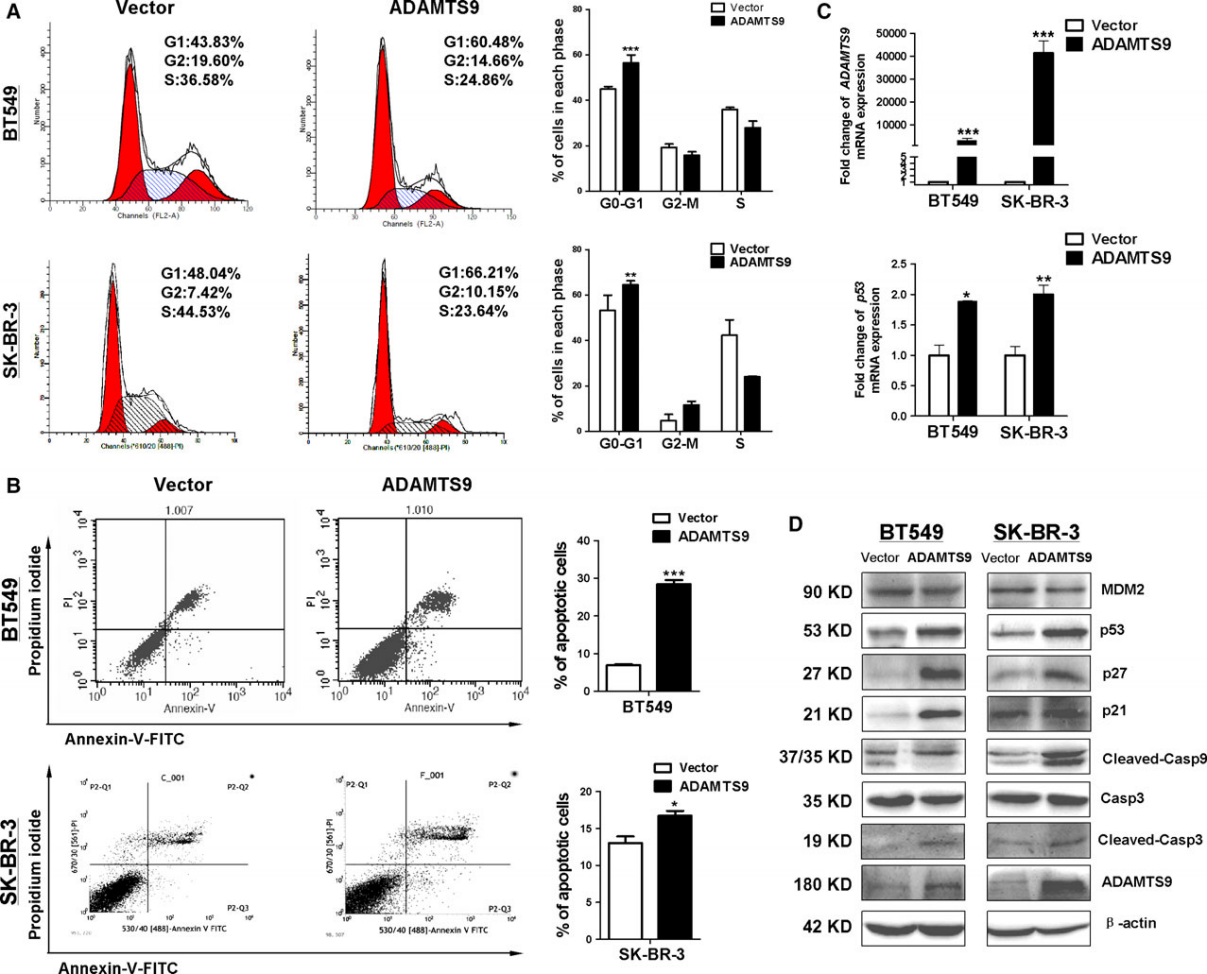
ADAMTS9 induces cell cycle arrest at the G0/G1 phase and cell apoptosis in and BT549 and SK‐BR‐3 cells. (**A**) Effect of cell cycle distribution of vector‐ and ADAMTS9‐transfected BT549 and SK‐BR‐3 cells was detected by flow cytometry analysis. Representative flow cytometry plots (left) and histograms of cell cycle alterations (right). (**B**) Apoptosis of vector‐ and ADAMTS9‐transfected BT549 and SK‐BR‐3 cells was detected by Annexin V‐FITC/PI staining. Representative flow cytometry plots (left) and histograms of cell cycle alterations (right). (**C**) qRT‑PCR quantitatively confirmation of ectopic transcript expression of ADAMTS9 in vector‐ and ADAMTS9‐transfected BT549 and SK‐BR‐3 cells (top). And ectopic ADAMTS9 up‐regulates the expression of p53 detecting by qRT‐PCR in vector‐ and ADAMTS9‐transfected BT549 and SK‐BR‐3 cells (bottom). (**D**) Protein expression levels of MDM2, p53 and downstream molecules in vector‐ and ADAMTS9‐transfected BT549 and SK‐BR‐3 cells. *P < 0.05, **P < 0.01, ***P < 0.001.

To elucidate that *ADAMTS9* functions as a tumour suppressor, cell cycle distribution was detected. Flow cytometry analysis revealed accumulation of cells in the G0/G1 phase (increased from 45.08% in vector‐transfected to 56.4% in *ADAMTS9*‐transfected BT549 cells, *P* < 0.001; and from 53.24% in vector‐transfected to 64.43% in *ADAMTS9*‐transfected SK‐BR‐3 cells; *P* < 0.01; Fig. 4A). Additionally, Annexin V‐FITC/PI staining assay was performed. Compared to controls, an increase in the percentage of apoptotic cells was observed in *ADAMTS9*‐transfected BT549 (from 6.89% to 28.47%, *P* < 0.001) and SK‐BR‐3 cells (from 13.03% to 16.74%, *P* < 0.05; Fig. 4B), respectively, suggesting that *ADAMTS9* promotes apoptosis.

To explore the detailed mechanisms underlying *ADAMTS9*‐induced cell cycle arrest and apoptosis, the expression levels of cell cycle and apoptosis markers were examined in vector‐ and ADAMTS9‐transfected BT549 and SK‐BR‐3 cells using qRT‐PCR and Western blot. The results showed that p53 expression was significantly increased at both mRNA and protein levels following overexpression of *ADAMTS9* in both cell lines (Fig. 4C and D). Furthermore, the downstream cell cycle regulatory proteins, p21 and p27, were up‐regulated in *ADAMTS9*‐transfected cells, compared to the corresponding vector‐transfected cells. In the *ADAMTS9*‐transfected cells, the expression of MDM2, an important negative regulator of p53, was lower than that in vector‐transfected cells (Fig. 4D). To further address the apoptotic effect of *ADAMTS9* in breast cancer cells, Western blot was performed, which showed that ectopic *ADAMTS9* promoted the cleavage of caspase‐9 and caspase‐3 in both BT549 and SK‐BR‐3 cells (Fig. 4D).

### Ectopic ADAMTS9 expression inhibits cell migration and invasion and regulates EMT‐related molecules

**Figure 5:**
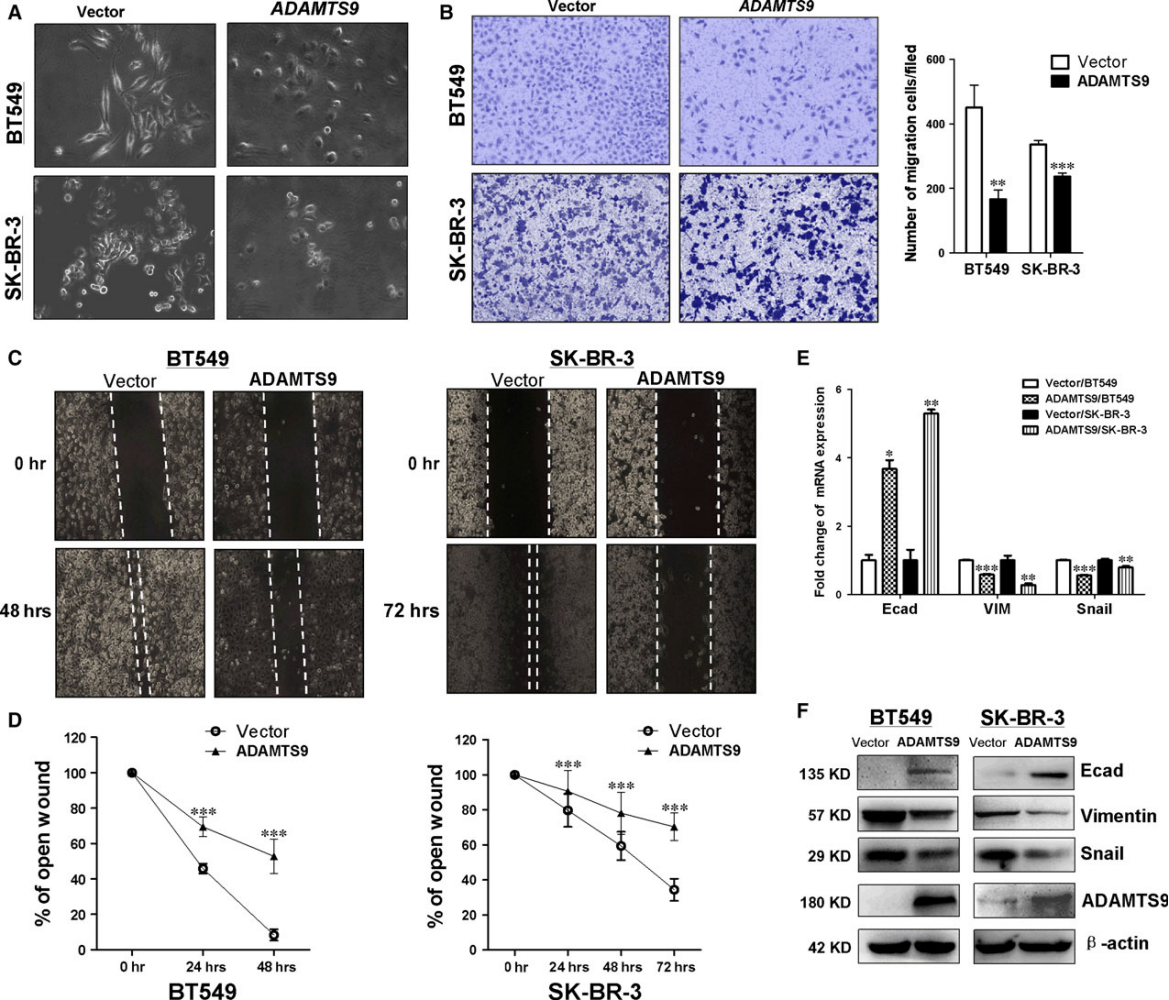
Ectopic expression of ADAMTS9 inhibits migration of BT549 and SK‐BR‐3 cells. (**A**) Morphology changes of BT549 and SK‐BR‐3 cells transfected with empty vectors or ADAMTS9 by phase‐contrast microscopy (Magnification, ×200). (**B**) Cell motility of vector‐ and ADAMTS9‐transfected cells (BT549 and SK‐BR‐3) were tested by transwell cell migration assays. Results from three independent experiments were quantified as mean ± S.D. (**C**) Cell motility of vector‐ and ADAMTS9‐transfected cells (BT549 and SK‐BR‐3) were tested by wound healing assays. Representative images were shown from three independent experiments. (**D**) Line charts of wound healing assays. The width of the remaining open wound was measured in relation to time 0 hr. (**E**) mRNA expression levels of E‐cadherin, VIM and SNAIL were examined by qRT‐PCR in vector‐ and ADAMTS9‐transfected cells (BT549 and SK‐BR‐3). (**F**) Protein expression levels of E‐cadherin, VIM and SNAIL in vector‐ and ADAMTS9‐transfected BT549 and SK‐BR‐3 cells. *P < 0.05, **P < 0.01, ***P < 0.001.

To establish whether *ADAMTS9* suppresses cell migration, we performed transwell cell migration assays. An obvious reduction in the number of migrated cells with ectopic expression of *ADAMTS9*, compared to control cells (*P* < 0.01 in BT549, *P* < 0.001 in SK‐BR‐3; Fig. 5B). Consistently, similar effect of *ADAMTS9* was observed by wound healing assay. Compared to control cells, *ADAMTS9‐*transfected cells delayed closure of wound gaps observed at 24 hrs (*P* < 0.001, Fig. 5C). The results indicate that *ADAMTS9* can effectively inhibit migration of BC cells.

**Figure 6:**
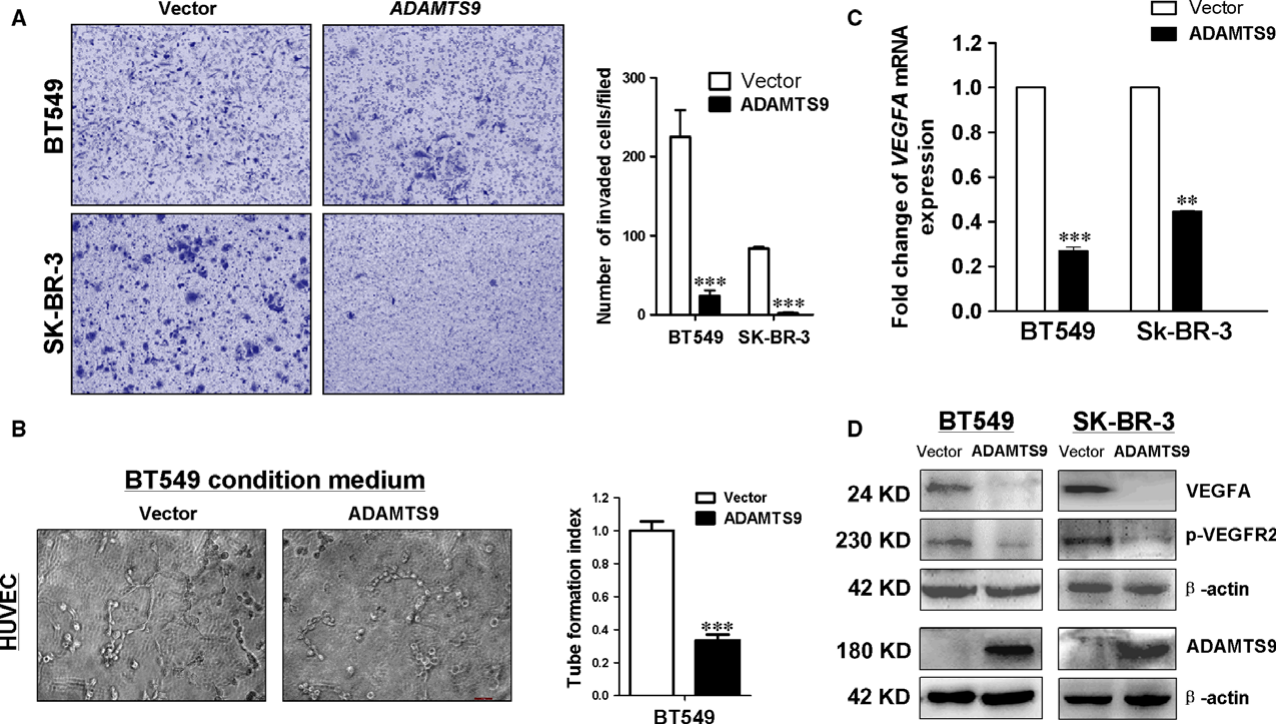
Ectopic expression of ADAMTS9 inhibits invasion of BT549 and SK‐BR‐3 cells, and suppresses angiogenesis *in vitro*. (**A**) Cell motility of vector‐ and ADAMTS9‐transfected cells (BT549 and SK‐BR‐3) were tested by transwell cell invasion assays. Results from three independent experiments were quantified as mean ± S.D. (**B**) Conditioned media from vector‐ and ADAMTS9‐transfected BT549 cells suppressed HUVEC tube formation. (**C**) mRNA expression levels of VEGFA were examined by qRT‐PCR in vector‐ and ADAMTS9‐transfected cells (BT549 and SK‐BR‐3). (**D**) Protein expression levels of VEGFA and p‐VEGFR2 in vector‐ and ADAMTS9‐transfected cells (BT549 and SK‐BR‐3). **P < 0.01, ***P < 0.001.

Next, we performed the cell invasion assay to investigate whether *ADAMTS9* suppresses breast cancer cell invasion using transwell cell invasion assays. After incubation for 48 hrs, the *ADAMTS9*‐transfected cells showed lower invasion ability than vector‐transfected cells (*P* < 0.001; Fig. 6A), clearly indicating a significant inhibitory effect of ectopic *ADAMTS9* on BC cell invasion.

We found that vector‐transfected cells exhibited spindle‐shaped morphology under the microscope. However, the majority of *ADAMTS9*‐transfected cells were elliptical, suggesting the possibility that *ADAMTS9* suppresses epithelial–mesenchymal transition (EMT) in breast cancer cells (Fig. 5A). Thus, we examined EMT markers in vector‐transfected and *ADAMTS9*‐transfected BT549 and SK‐BR‐3 cells by qRT‐PCR and Western blot assays. SNAIL and VIM expression was decreased, while E‐cadherin expression was increased in *ADAMTS9*‐transfected cells relative to vector‐transfected cells (Fig. 5E and F), suggesting that *ADAMTS9* suppresses EMT.

### ADAMTS9 inhibits angiogenesis *in vitro*

*ADAMTS9* has been identified as an angiogenesis inhibitor in several cancer types 14, 29. Accordingly, we speculated that *ADAMTS9* inhibits carcinogenesis in breast cancer, at least in part, through blocking angiogenesis. We performed the HUVEC tube formation assay to examine the effect of *ADAMTS9* on angiogenesis of BC cells *in vitro*. The result showed that conditioned culture medium from *ADAMTS9*‐transfected BT549 cells dramatically reduced the tube‐forming ability of HUVECs in Matrigel (Fig. 6B, *P* < 0.001). To further investigate the mechanisms underlying the anti‐angiogenic effects of *ADAMTS9*, the expression of vascular endothelial growth factor A (VEGFA), a key angiogenesis‐related factor, was examined. As expected, VEGFA mRNA and protein expression was significantly down‐regulated by *ADAMTS9*. Subsequently, reduced phospho‐VEGF Receptor‐2 (Tyr1175) expression was also detected in *ADAMTS9*‐transfected cells, compared to the vector‐transfected BT549 and SK‐BR‐3 cells (Fig. 6C and D).

### ADAMTS9 inhibits the PI3K/AKT pathway *via* modulation of EGFR and TGFβ

**Figure 7:**
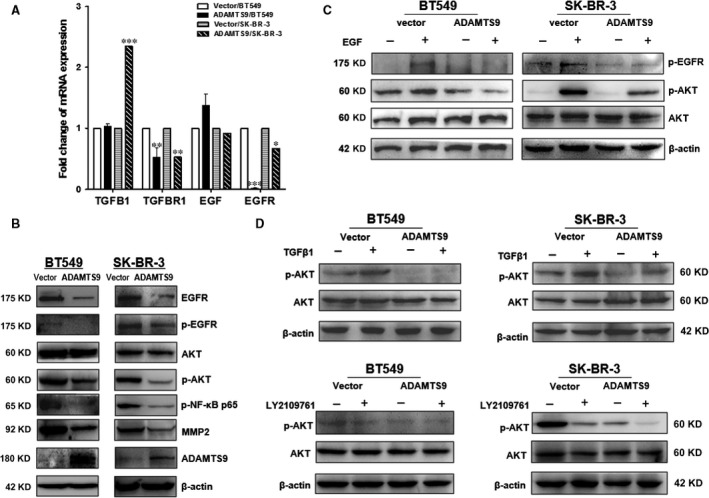
ADAMTS9 inhibits the activities of AKT through interacting with EGFR and TGFβ1/TβR(I/II). (**A**) mRNA expression levels of TGFβ1, TβRI, EGF and EGFR were examined by qRT‐PCR in vector‐ and ADAMTS9‐transfected cells (BT549 and SK‐BR‐3). (**B**) Protein expression levels of EGFR, p‐EGFR, AKT, p‐AKT, p‐NFκB p65, MMP2 signal pathway in vector‐ and ADAMTS9‐transfected BT549 and SK‐BR‐3 cells. (**C**) The expression of p‐EGFR and p‐AKT after EGF treatment on vector‐ and ADAMTS9‐transfected BT549 and SK‐BR‐3 cells. (**D**) The expression of p‐AKT after TGFβ1 or LY2109761 treatment on vector‐ and ADAMTS9‐transfected BT549 and SK‐BR‐3 cells. *P < 0.05, **P < 0.01, ***P < 0.001.

While previous studies have demonstrated that *ADAMTS9* inhibits the AKT/mTOR signalling pathway 14, the underlying mechanisms remain unknown. To determine the active site of *ADAMTS9*, we focused on two key upstream molecules of the AKT pathway, EGFR and TGFβ. Expression of *TGF*β*1*,* T*β*RI*,* EGF* and *EGFR* mRNA was examined using qRT‐PCR. *TGF*β*1* and *EGF* mRNA levels were up‐regulated or remained unchanged in *ADAMTS9*‐transfected BT549 and SK‐BR‐3 cells, respectively, compared to that in the vector‐transfected cells. In contrast, *TGF*β*RI* and *EGFR* were dramatically down‐regulated by *ADAMTS9* (Fig. 7A). Western blot results showed that ectopic *ADAMTS9* suppressed expression of EGFR, phosphorylated EGFR (Tyr‐1068), phosphorylated AKT (Ser473), phosphorylated NFκB p65 (Ser536) and MMP2 protein expression (Fig. 7B).

The vector‐ or ADAMTS9‐transfected BT549 and SK‐BR‐3 cells were treated with EGF (50 ng/ml) for 40 min., and protein levels of phosphorylated EGFR (Try1068), AKT, phosphorylated AKT (Ser473) and β‐actin (as the loading control) were determined by Western blot assays. Compared to the vector‐transfected cells, the *ADAMTS9‐*transfected cells showed significantly lower phosphorylation levels of EGFR and AKT after treatment with EGF, suggesting that *ADAMTS9* inhibited the AKT pathway *via* inhibiting phosphorylation of EGFR (Fig. 7C). The vector‐ or *ADAMTS9*‐transfected BT549 and SK‐BR‐3 cells were also treated with TGFβ1 (1 ng/ml, 24 hrs) or LY2109761 (selective TGF‐β receptor type I/II dual inhibitor, 5 μM, 24 hrs); the expression of AKT, phosphorylated AKT (Ser473) and β‐actin (as the loading control) was detected by Western blot. Compared to the vector‐transfected cells, the *ADAMTS9*‐transfected cells showed significantly lower phosphorylation levels of AKT after treatment with TGFβ1 (Fig. 7D). The negative effect of LY2109761 on phosphorylation of AKT was enhanced by *ADAMTS9* (Fig. 7D). The results indicate that *ADAMTS9* suppresses AKT activation induced by TGFβ1.

## Discussion

Matrix metalloproteinases facilitate cancer dissemination through degrading extracellular matrix components, which promotes cancer cell invasion and metastasis 30, 31, 32. However, lately researches have exposed that multiple members of the ADAMTS family exhibit tumour suppressor properties 8. For instance, *ADAMTS1, ADAMTS9* and *ADAMTS18* are frequently silenced by methylation in several cancers, suggesting them as potential tumour suppressors 10, 11, 12, 13, 14, 33, 34, 35, 36, 37.

In this study, we show for the first time that *ADAMTS9* is epigenetically silenced in both BC cell lines and primary breast tumours, but remains unmethylated in normal breast tissues and cells. The reduced *ADAMTS9* expression is closely associated with promoter methylation. Demethylation treatment significantly restored *ADAMTS9* expression, indicating promoter methylation could be the main mechanism underlying *ADAMTS9* inactivation in breast cancer. Furthermore, ectopic expression of *ADAMTS9* in BC cells inhibited proliferation, arrested cell cycle at the G0/G1 phase, enhanced apoptosis and reduced tube formation ability of HUVECs. These results suggest that *ADAMTS9* is a potential TSG in breast cancer that is silenced by promoter methylation.

Consistent with reports in other cancer types 14, we found *ADAMTS9* inhibits the AKT/mTOR signalling pathway in breast cancer cells. The AKT signalling pathway is commonly hyperactive in cancers, which promotes tumour progression involving various downstream molecules such as MDM2, p21, p27 and NF‐κB 38. As the most important tumour suppressor, p53 arrests cell cycle and induces apoptosis, thereby inhibiting cancer development 39, 40. AKT promotes p53 degradation through MDM2‐mediated ubiquitination 41. Our results showed that *ADAMTS9* markedly up‐regulated p53 and inactivated AKT, suggesting *ADAMTS9‐*inhibited cancer cell proliferation may involve AKT suppression and p53 activation.

How AKT is suppressed by *ADAMTS9* is elusive. Because EGFR is a strong upstream activator of the AKT pathway 42, 43 and is influenced by other members of the ADAMTS family such as ADAMTS1 and ADAMTS8 44, 45, we examined and found that ADAMTS9 can reduce the phosphorylated level of EGFR and suppress EGFR ligand‐induced AKT activation in breast cancer cells. Because ADAMTS9 suppressed TGFβ1 expression and TGFβ1 can activate the AKT pathway 46, 47, 48, 49, 50, we also examine the role of TGFβ1 in ADAMTS9's inhibitory effect on AKT. Our results suggest that ADAMTS9 inhibits TGFβ1‐induced AKT activation, which may involve TβR(I/II) in breast cancer cells. It should be noted that there is crosstalk between TGFβ1 and EGFR 51, whether the interplay of these two pathways is involved in the ADAMTS9‐mediated AKT suppression needs further studies.

We show *ADAMTS9* effectively suppressed the angiogenic function in breast cancer cells, which is consistent with reports on gastric, esophageal and nasopharyngeal carcinoma 14, 29. Additionally, our findings indicate that VEGFA and phosphorylated VEGFR2 were suppressed by ADAMTS9. It is reported that the AKT/mTOR pathway up‐regulates HIF1α, which is the upstream regulator of VEGFA 52. Whether ADAMTS9 suppresses breast cancer angiogenesis involving HIF1α suppression deserves further studies.

In conclusion, we provide evidence suggesting *ADAMTS9* as a novel tumour suppressor gene that is silenced by promotor hypermethylation in breast cancer. ADAMTS9 exerts its tumour suppressor activity by inhibiting cell proliferation, colony formation, arresting the cell cycle, inducing apoptosis, suppressing angiogenesis and suppressing EMT. Furthermore, ADAMTS9 inhibits the AKT pathway through suppressing EGFR and TGFβ1/TβR(I/II) in breast cancer cells.

## Consent for publication

We confirm that this manuscript is original. If accepted, the article will not be published elsewhere in the same form, in any language, without the written consent of the publisher.

## Competing interests

The authors declare no conflict of interest.

## Funding source

This study was supported by the Chongqing Natural Science foundation (cstc2016jcyjA0309), National Natural Science Foundation of China (#81572769, # 81302307) and VC special research fund from The Chinese University of Hong Kong.
